# Sulfur-Containing Amino Acid Homeostasis in the Central Nervous System: From Physiology Regulation to Metal-Induced Neurotoxicity

**DOI:** 10.3390/metabo16070461

**Published:** 2026-07-01

**Authors:** Wendy Leslie González-Alfonso, Gustavo Ignacio Vázquez-Cervantes, Itamar Flores, María E. Gonsebatt, Gonzalo Pérez de la Cruz, Saúl Gómez Manzo, Aleli Salazar, Benjamín Pineda, Verónica Pérez de la Cruz

**Affiliations:** 1 Neuroimmunology Laboratory, National Institute of Neurology and Neurosurgery “Manuel Velasco Suárez”, Mexico City 14269, Mexico; wendy.gonzalez@innn.edu.mx (W.L.G.-A.); gustavo.vazquez@innn.edu.mx (G.I.V.-C.); itamar.flores@innn.edu.mx (I.F.); aleli.salazar@innn.edu.mx (A.S.); 2 Sistema Nacional de Investigadoras e Investigadores, Secretaría de Ciencia, Humanidades, Tecnología e Innovación, Mexico City 03940, Mexico; mgonsebatt@gmail.com; 3 Department of Mathematics, Faculty of Sciences, Universidad Nacional Autónoma de México (UNAM), Mexico City 04510, Mexico; gonzalo.perez@ciencias.unam.mx; 4 Laboratorio de Bioquímica Genética, Instituto Nacional de Pediatría, Secretaría de Salud, Mexico City 04530, Mexico; saulmanzo@ciencias.unam.mx; 5 Neurobiochemistry and Behavior Laboratory, National Institute of Neurology and Neurosurgery “Manuel Velasco Suárez”, Mexico City 14269, Mexico

**Keywords:** sulfur-containing amino acid, neurological dysfunction, metal exposure, glutathione

## Abstract

Sulfur-containing amino acids (SCAA) and their metabolites constitute an integrated metabolic network essential for central nervous system (CNS) function. In mammals, sulfur metabolism links one-carbon metabolism, the methionine cycle and the transsulfuration pathway, thereby connecting nutrient availability with redox regulation, methylation reactions, neurotransmitter synthesis and cellular adaptation to stress. Among these metabolites, methionine, cysteine, glutathione, taurine, homocysteine and hydrogen sulfide play key roles in neuronal physiology, mitochondrial homeostasis, synaptic plasticity and antioxidant defense. Alterations in SCAA metabolism have been increasingly associated with neurological and neurodevelopment disorders, which share common features such as oxidative stress, mitochondrial dysfunction, altered glutamatergic signaling, impaired methylation capacity and neuroinflammation. These pathological mechanisms are also observed following exposure to toxic metals, suggesting the existence of convergent pathways between environmental neurotoxicity and neurological diseases. Several studies showed that chronic exposure to arsenic, mercury, cadmium, lead, and other toxic metals disrupts sulfur amino acid homeostasis by affecting methionine remethylation, transsulfuration activity, glutathione synthesis and reactive sulfur species production. Due to sulfur-containing metabolites possessing antioxidant and metal-binding properties, these pathways are also involved in adaptive detoxification response. However, sustained disruption of sulfur metabolism may compromise neuronal resilience and increase vulnerability to neurological dysfunction. This narrative review integrates current evidence on the physiological roles of SCAA in the CNS, and examines how toxic metals disrupt sulfur metabolic pathways. By combining findings from experimental studies, human data and exploratory transcriptomic analyses, we propose that disruption of SCAA homeostasis represents a mechanistic link between environmental metal exposure and increased vulnerability to neurological disease.

## 1. Introduction

Sulfur-containing amino acids (SCAAs), particularly methionine (Met), cysteine (Cys), and homocysteine (Hcy), together with sulfur-related metabolites such as glutathione (GSH), taurine, S-adenosylmethionine (SAM), and hydrogen sulfide (H_2_S), among others, play essential roles in cellular metabolism. These compounds are involved in redox homeostasis, detoxification, methyl-group transfer and cellular signaling pathways [1]. In the central nervous system (CNS), their relevance is particularly evident because they contribute to antioxidant defense, mitochondrial activity and neuronal homeostasis. Recent evidence indicates that disturbances in SCAA metabolism are associated with neurodegeneration, neurodevelopmental disorders, and other brain dysfunctions [2,3,4].

Among SCAA, Cys is especially important due to the reactivity of its thiol group. This property allows Cys to participate in redox reactions, protein folding, metal binding, and antioxidant responses [1,5,6]. Cys is also the rate-limiting precursor for GSH (ϒ-L-glutamyl-cysteine-glycine), the most abundant intracellular antioxidant in the brain. GSH depends on Cys availability and is required to neutralize reactive oxygen species (ROS) and reactive nitrogen species (RNS), to maintain protein thiol balance, and to support detoxification pathways mediated by GSH-dependent enzymes [7]. Beyond antioxidant defense, sulfur-containing metabolites are also central to cellular adaptation under stress conditions. GSH participates in the conjugation and elimination of xenobiotics and their metabolites [7], while the transsulfuration pathway links methionine metabolism to endogenous Cys synthesis [8,9]. Activation of these pathways becomes particularly relevant during toxic or inflammatory insults, when cellular demand for antioxidant protection increases [3,10].

Exposure to toxic metals represents another condition in which sulfur metabolism may be critically involved. Metals such as mercury (Hg), cadmium (Cd), and lead (Pb), as well as the metalloid arsenic (As), can induce oxidative stress, mitochondrial dysfunction, abnormal calcium signaling and neuronal death [11]. Many of these processes converge with mechanisms implicated in neurodegenerative and neurodevelopmental disorders, suggesting that environmental exposure may contribute to disease progression or susceptibility [12,13,14]. Because sulfur-containing compounds have both redox-active and metal-binding properties, alterations in SCAA homeostasis may influence the biological response to metal exposure. Consistent with this idea, altered levels of GSH, Cys, Hcy, taurine, and related metabolites have been reported in exposed populations, as well as in patients with neurological disorders. This narrative review integrates current evidence on the role of SCAA in CNS physiology and examines how toxic metals disrupt sulfur metabolic pathways. In addition, we incorporate exploratory analysis of publicly available transcriptomic datasets to characterize the dynamic of expression patterns of SCAA-related genes in the brain. We discuss SCAA relevance to CNS physiology and pathology, with particular emphasis on the hypothesis that metal-induced disruption of SCAA homeostasis may compromise neuronal resilience and increase susceptibility to neurological diseases.

## 2. Sulfur-Containing Amino Acid Homeostasis and Physiological Roles

### 2.1. Sulfur-Containing Amino Acids (SCAA)

SCAAs comprise a group of amino acids and sulfur-related metabolites that are essential for cellular structure, intermediary metabolism and redox regulation. Their physiological availability depends on dietary intake, endogenous biosynthesis, tissue-specific transport systems, and metabolic demand [8,15]. Nutritional sources rich in SCAA include animal-derived proteins such as meat, poultry, fish/seafood, milk, cheese, and eggs, as well as plant-derived sources including soybeans, nuts, seeds, garlic, onions and cruciferous vegetables [15]. Since sulfur metabolism is closely linked to protein turnover and antioxidant defense, dietary supply can significantly influence systemic and tissue homeostasis.

The primary proteinogenic SCAAs are Met and Cys [1]. Met is an essential amino acid that must be obtained from the diet, whereas Cys can be synthesized endogenously from Met through the transsulfuration pathway (TSP). Beyond their incorporation into proteins, both amino acids serve as precursors for a wide range of sulfur-containing metabolites with critical biological functions.

Met takes up a central metabolic position since it is the precursor of SAM, the major methyl group donor in mammalian cells. Through SAM-dependent transmethylation reactions, Met metabolism contributes to DNA methylation, phospholipid synthesis, neurotransmitter metabolism, and epigenetic processes involved in development and cellular adaptation [16]. Therefore, alterations in Met availability or methylation capacity may affect gene regulation and neural function.

Cys is also important since the sulfhydryl (-SH) group of this amino acid confers redox and nucleophilic properties. Cys residues stabilize protein conformation through disulfide bond formation, participate in catalytic sites of enzymes, and serve as a sensor of the intracellular redox environment [5,6]. In addition, Cys is the rate-limiting precursor of GSH [7]. Through GSH synthesis, Cys availability directly influences peroxide detoxification, maintenance of protein thiol status and cellular resistance to oxidative stress [7].

Other sulfur-related metabolites like cystathionine, lanthionine, homolanthionine, thiocysteine, and H_2_S are byproducts of SCAA metabolism and have emerged as biologically relevant products of sulfur metabolism, particularly under conditions of altered redox state [17,18,19]. Collectively, SCAAs form an integrated metabolic network that extends far beyond protein synthesis. Their functions include antioxidant defense, methyl-group transfer, detoxification, neurotransmission support, mitochondrial function and adaptation to cellular stress, making them especially relevant to tissues with high metabolic activity such as the brain.

### 2.2. SCAA Homeostasis

SCAA homeostasis is tightly regulated by dietary uptake, cellular transport, intercellular synthesis, SCAA metabolism and their tissue biodistribution. Due to SCAA requirements differing tissues, sulfur metabolites display marked compartmentalization across organs and cell types. Particularly in CNS, access to circulating amino acids is restricted by the blood–brain barrier (BBB), while neurons and glial cells show distinct SCAA metabolic capacities [20,21]. Thus, in the CNS maintenance of SCAA homeostasis depends on coordinated interaction among endothelial cells, astrocytes, neurons and choroid plexus, highlighting the importance of metabolic compartmentalization within the neurovascular unit.

Transport of SCAA across cellular membranes is mediated primarily by members of the solute carrier (SLC) superfamily. Among these, sodium-dependent SLC1 and the sodium-independent SLC7 transporter families are the main carriers for most of the SCAA [22,23]. Within the SLC1 family, the XAG and ASC system are involved in Cys transport, whereas in the SLC7 family, L and y^+^L systems contribute to Met and Hcy transport by neutral/cationic amino acid exchange with other amino acids. The cystine:glutamate antiporter systems are Xc- and b0, + [22,23].

Transporters such as the SLC7A5 (LAT1, from L system), SLC7A6 (from y^+^L system), SLC7A11 (xCT, from Xc- system), SLC1A1 (EAAT3/EAAC1, from X-AG system) and SLC1A4 (ASCT1, from ASC system) have been involved in nutrient delivery across the BBB, neuronal amino acid uptake, glial metabolic support and neurodevelopment processes [21,24,25,26]. Dysregulation of these transport system may therefore alter SCAA availability and compromise redox balance or neurotransmitter metabolism [22]. The cystine:glutamate antiport Xc-, whose light chain is encoded by SLC7A11, is particularly important since it imports cystine in exchange for glutamate. Once inside the cell, cystine is rapidly reduced to Cys, thereby supporting intercellular Cys availability for GSH synthesis and other metabolic processes.

In contrast, taurine transport is carried out mainly by members of the sodium and chloride-dependent SLC6 amino acid transporter family, such as SLC6A6 (TauT), which is widely expressed in excitable tissues, including the brain and retina [27]. Given that transport capacity, substrate competition, oxidative state and nutritional status can all modify intracellular amino acid pools, membrane transport represents a critical level of regulation in sulfur metabolism.

#### 2.2.1. SCAA Metabolism

**Figure 1:**
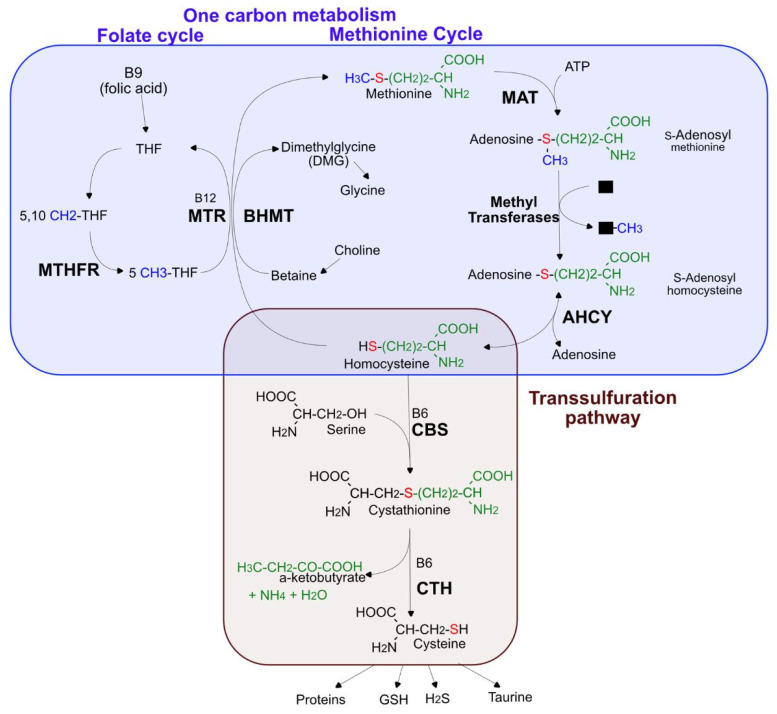
Sulfur-containing amino acids (SCAAs) metabolism linking one-carbon metabolism with the transsulfuration pathway (TSP). One-carbon metabolism involves the folate and methionine cycles, which mediate methyl-group transfer reactions, whereas the transsulfuration pathway directs sulfur flux from homocysteine (Hcy) to cysteine (Cys) and downstream metabolites such as glutathione (GSH). Atoms are color-coded to indicate their flux in the metabolic reactions. Dark squares represent any metabolite subject to methylation by methyltransferases. The direction of the arrows indicates the metabolic flux. Metabolites: THF: tetrahydrofolate, 5-CH_3_-THF: 5-methyltetrahydrofolate, 5,10-CH_2_-THF: 5-10-methylenehydrofolate, ATP; adenosine triphosphate, B6: pyridoxal 5′-phosphate, B12: cobalamin, GSH: glutathione. Enzymes: MTHFR: Methyl-tetrahydrofolate reductase, MTR: methionine synthase, BHMT: betaine-homocysteine methyltransferase, MAT: methionine adenosyl transferase, AHCY: S-adenosylhomocysteine hydrolase, CBS: cystathionine β-synthase, CTH: cystathionine γ-lyase.

The conversion of Met to Cys encompasses two sequential pathways, the Met cycle and the transsulfuration pathway (TSP) (Figure 1) [8]. Together, these pathways link methyl-group metabolism with antioxidant defense and sulfur redistribution. The Met cycle involves the conversion of Met to SAM by methionine adenosyltransferase (MAT). SAM then serves as a methyl donor for numerous methyltransferase reactions, which lead to S-adenosylhomocysteine (SAH). SAH is hydrolyzed by S-adenosylhomocysteine hydrolase (SAHH, also known as AHCY) to produce homocysteine (Hcy) [8]. Hcy can be remethylated to Met through two major reactions: methionine synthase (MTR) catalyzes folate and vitamin B12-dependent remethylation using 5-methyl tetrahydrofolate as methyl donor, whereas betaine–homocysteine methyltransferase (BHMT) uses betaine as the methyl source, thus completing the Met cycle [8]. These reactions also constitute part of the one-carbon metabolism pathway [8].

Alternatively, Hcy can also enter the TSP, an irreversible route that channels sulfur from Met toward Cys synthesis [28]. In the classical TSP, the enzyme cystathionine β-synthase (CBS) condenses Hcy with serine to form cystathionine [28]. Cystathionine is then cleaved by cystathionine γ-lyase (CTH, also known as CSE) to yield Cys, α-ketobutyrate and ammonia. The role of Hcy as a substrate for both the Met cycle and the TSP makes this amino acid an essential molecule for SCAA metabolism [8]. Therefore, Hcy is a central metabolic node that determines the partitioning of sulfur flux between methylation reactions and antioxidant defense.

The Cys generated has multiple metabolic fates. It may be incorporated into protein, used for coenzyme A synthesis, converted to taurine or utilized to produce H_2_S (a gaseous signaling molecule with neuromodulatory and cytoprotective properties) (Figure 1) [1,8]. Most importantly, Cys is required for GSH synthesis through the sequential actions of glutamate–cysteine ligase and glutathione synthetase.

The relative contribution of *de novo* Cys synthesis versus extracellular uptake varies by tissue. In the liver, transsulfuration is a major source of Cys for GSH production, whereas in the brain, many cells depend strongly on extracellular cysteine/cystine transport. SCAA metabolism provides approximately 50% of the Cys incorporated into GSH in the liver and around 30% in astrocytes [10,28]. Astrocytes appear to retain a greater capacity for sulfur amino acid interconversion than neurons, which may partially explain their central role in supporting neuronal antioxidant defenses [29]. Under oxidative stress, activation of transsulfuration and cystine uptake pathways can increase Cys availability, thereby sustaining GSH synthesis and enhancing resistance to cellular damage [10,28].

Although the biochemical pathways governing SCAA metabolism are well characterized, their organization within the CNS is highly dynamic and exhibits marked temporal, regional and cell-specific specialization. Understanding the dynamics of SCAA-related genes during developmental states and throughout life may provide insight into differential metabolic vulnerabilities associated with environmental and neurological disorders.

#### 2.2.2. Genes Involved in SCAA Homeostasis Are Differentially Expressed Across the Brain

The metabolic requirements of the brain change substantially throughout development and aging. Since SCAA metabolism supports redox homeostasis, methylation capacity and neurotransmission, we hypothesized that genes involved in these pathways exhibit dynamic expression patterns across brain regions and during different stages of life. To explore this possibility, we performed exploratory analyses using publicly available single-cell and bulk RNA sequencing datasets. These analyses were not intended to establish causality but rather to provide a spatial and temporal framework for interpreting the physiological and pathological roles of SCAA metabolism in the brain.

**Figure 2:**
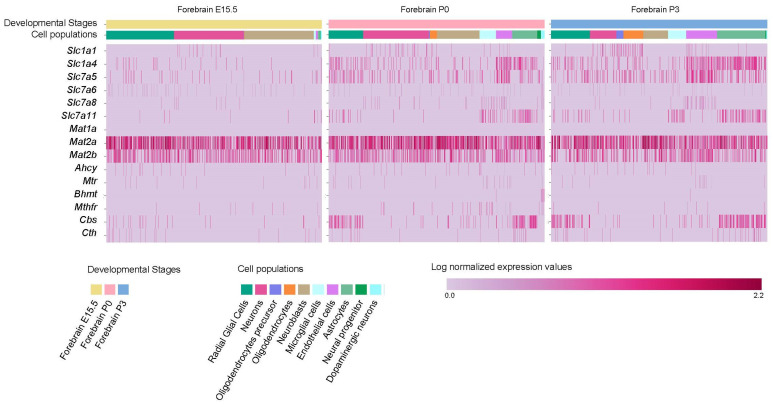
Developmental and cell-type-specific expression of SCAA-related genes in the mouse brain. Public single-cell mRNA sequencing data (GSE133531) from embryonic day (E) 15.5, and postnatal day (P) 0, and P3 [30] were analyzed using the TrailMaker platform: https://app.trailmaker.parsebiosciences.com/ (accessed on 27 November 2025). The heatmap displays the Log-normalized expression values of SCAA-related genes (*Slc1a1*, *Slc1a4*, *Slc7a5*, *Slc7a6*, *Slc7a8*, *Slc7a11*, *Mat1a*, *Mat2a*, *Mat2b*, *Ahcy*, *Mtr*, *Bhmt*, *Mthfr*, *Cbs* and *Cth*) of each cell grouped by developmental stages and brain cell population. Proportion of cell population within each stage is represented by the width of the bar. Cell count analyzed for stages: *E15.5 (9052 cells)*, *P0 (4686 cells)*, and *P3 (3827 cells).*

Genes involved in SCAA transport and metabolism show marked spatial, temporal, and cell type-specific expression patterns in the developing brain. To further examine how these pathways may be organized within the CNS, an exploratory analysis of publicly available single-cell RNA sequencing data from mouse brain during development (GSE133531), from E15.5 to early postnatal days (P3), was performed (Figure 2, Supplementary Material Section S1.2.2) [30,31]. Our aim was to characterize how the genes related to SCAA homeostasis are distributed across brain cell populations and how their expression changes during development. Overall, SCAA transporter genes increase over time, especially *Slc1a4*, *Slc7a5* and *Slc7a11*, with a more pronounced rise during the early postnatal period, consistent with the increasing demands associated with gliogenesis, synaptogenesis and cell maturation [32,33].

Among the genes analyzed, *Mat2a* and *Mat2b* showed broad expression across different brain cell populations and consistently ranked among the most abundant SCAA-related transcripts throughout development, especially at P0 (Figure 2). There are two isoforms of MAT: MAT1 and MAT2. MAT2 proteins are encoded by *Mat2a* (catalytic subunit) and *Mat2b* (regulatory subunit). It is known that the MAT2 complex shows a lower Km for Met than MAT1 and is sensitive to SAM inhibition [34]. The results in Figure 2 are in accordance with the predominant role of MAT2 in extrahepatic tissues, including the brain, where it catalyzes the synthesis of SAM, the universal methyl donor [34]. The sustained expression of *Mat2a*/*Mat2b* during development likely reflects the continuous requirement for methylation reactions during neural maturation [35].

The BBB endothelial cells showed important SCAA transporters expression, which is consistent with the central role of the BBB in regulating amino acid entry into the CNS. Brain endothelial cells express a specialized repertoire of transporters allowing selective uptake and exchange of circulating nutrients while preserving CNS homeostasis [21]. Distinct transport patterns were also evident among neural cell types. *Slc1a4* and *Slc7a5* were expressed ubiquitously among cell types, while *Slc7a8* and *Slc7a11* were mainly expressed in endothelial and glial cells, respectively. *Slc1a1* expression was higher in neurons and oligodendrocytes. These expression patterns suggest that SCAA handling is metabolically compartmentalized within the brain. Astrocytes appear equipped to utilize multiple sulfur sources, including cystine, Cys, and Met, whereas neurons are highly dependent on extracellular Cys supply and glial-mediated support. This idea is in line with previous evidence indicating that astrocytes play a central role in maintaining neuronal GSH precursor and redox buffering capacity [7,10]

Notably, expression of *Cbs* and *Cth* increased during development and reached higher levels during the early postnatal period in astrocytes and radial glial cells (Figure 2). In addition, remethylation enzymes, *Mtr* and *Bhtm*, expression levels were very low, suggesting that amino acid uptake and metabolic flux from Met to SAM and through TSP are more relevant during this period. This temporal profile suggests that transsulfuration may be particularly relevant during periods of rapid growth, synaptic remodeling and adaptation to postnatal oxidative metabolism.

**Figure 3:**
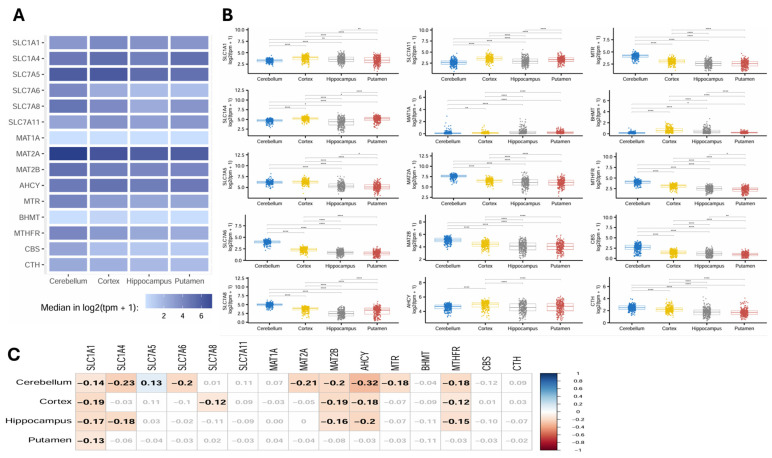
RNA-seq gene expression data (RSEM TPM) of SCAA-related genes (*Slc1a1*, *Slc1a4*, *Slc7a5*, *Slc7a6*, *Slc7a8*, *Slc7a11*, *Mat1a*, *Mat2a*, *Mat2b*, *Ahcy*, *Mtr*, *Bhmt*, *Mthfr*, *Cbs* and *Cth*) in adult human brain samples for four brain regions: cerebellum, cortex, hippocampus, putamen; tpm values were transformed using the log2(tpm + 1) transformation. (**A**) Heatmap of median expression values. (**B**) Comparison of expression levels of each SCAA-related gene across the four brain regions using the Kruskal–Wallis test (all *p* < 0.05), followed by Dunn’s post hoc test for multiple pairwise comparisons. Significant pairwise differences are denoted by * *p* < 0.05, ** *p* < 0.01, and **** *p* < 0.0001. (**C**) Heatmap showing significant (*p* < 0.05) Spearman correlations between gene expression levels and age, considering six standard 10-year age brackets. Gene expression and clinical data were obtained from the Genotype-Tissue Expression (GTEx) project through the GTEx Portal API using the R package gtexr (GTEx version 10) [38].

In addition to the developmental single-cell RNA sequencing analysis described above, we further explored the expression profile of SCAA-related genes in adult human brain tissues using publicly available RNA sequencing datasets from Genotype-Tissue Expression (GTEx) project (Figure 3, Supplementary Material Section S1.2.2) [36,37,38,39]. This complementary analysis allowed us to evaluate whether genes involved in sulfur metabolism display regional specialization in the mature human brain. Overall, most SCAA-related genes showed detectable expression levels across all regions, although important differences were observed (Figure 3A). The highest expression levels for several genes were found in the cerebellum and cortical regions, suggesting sulfur metabolism activity in areas characterized by high synaptic and energetic demand. Among transporter genes, *Slc1a1*, *Slc1a4*, *Slc7a5*, *Slc7a8* and *Slc7a11* showed intermediate to high expression across different brain regions, whereas *Slc7a6* and *S1c7a8* expressions were more enriched in the cerebellum (Figure 3B). These patterns are consistent with the heterogeneous distribution of amino acid transport systems within the CNS and support the existence of region-specific metabolic requirements.

Genes associated with Met cycle and methylation capacity, including *Mat2a*, *Mat2b* and *Ahcy,* were broadly expressed throughout the adult brain. Notably, *Mat2a* showed the highest overall expression among the SCAA-related genes analyzed, underscoring the importance of SAM synthesis for neural homeostasis and cellular maintenance in adult CNS. In contrast, remethylation-related genes, *Mtr* and *Mthfr*, showed comparatively lower expression across regions, with the highest expression in the cerebellum (Figure 3B). As expected for extrahepatic tissue, little expression of *Mat1a* or *Bhmt* was observed in the adult human brain. Nevertheless, experimental studies in animal models suggest that the BHMT-dependent pathway may still indirectly influence brain physiology through systemic one-carbon metabolism and methyl donor availability [40,41].

Interestingly, the correlation analysis between gene expression and age revealed a progressive decline in several SCAA-related transcripts across multiple brain regions with age (Figure 3C). Among the most consistent changes, the expression of *Slc1a1*, *Mat2b*, *Ahcy* and *Mthfr* negatively correlated with age in several regions. Although the functional implications of these changes remain unclear, they may reflect age-associated alterations in amino acid transport, methylation potential, and sulfur metabolic flexibility. Given the central role of sulfur metabolism in redox balance, mitochondrial function, and neurotransmission, these observations are consistent with the progressive decline in cognitive and cellular resilience observed during aging.

Distinct expression patterns were also observed for transsulfuration enzymes. *Cbs* was predominantly enriched in the cerebellum relative to other brain regions (Figure 3B), while *Cth* showed a broader distribution, and in some regions, similar or higher expression than *Cbs* in the adult brain (Figure 3A). These findings differ from those observed during early developmental stages (Figure 2), indicating that sulfur metabolic pathways undergo dynamic reorganization throughout brain maturation and aging. Previous studies in rodents similarly reported region-specific expression of CBS with abundance in the cerebellum and the hippocampus, as well as developmental changes in transsulfuration enzyme levels during postnatal maturation [42,43].

Taken together, these data reinforce the concept that SCAA homeostasis in the brain is highly compartmentalized and dynamically regulated across both development and adulthood. Rather than relying on a single metabolic route, sulfur metabolism appears to depend on coordinated interactions among amino acid transport systems, one-carbon metabolism, transsulfuration activity, and cell-type-specific metabolic coupling within distinct neural regions. This spatial and temporal organization may influence how different brain regions respond to environmental stressors and could contribute to the selective vulnerability observed in neurological disorders.

### 2.3. Role of SCAA in Brain Physiology

Due to the fact that the CNS has high metabolic demands and limited regenerative capacity, adequate SCAA availability is particularly important for maintaining neuronal viability and circuit integrity. As proteinogenic amino acids, Cys and Met are essential for protein synthesis, and thus they are key components involved in brain development and cellular processes that depend on protein translation, such as long-term memory consolidation [44]. Their importance extends beyond simple incorporation into polypeptides. Cys residues are critical determinants of protein conformation through disulfide bond formation, a process that stabilizes extracellular proteins, receptors, and secreted peptides [45,46,47]. In addition, cysteinyl thiols function as a regulatory switch for many enzymes, ion channels, transcription factors, and signaling proteins, allowing redox cues to be translated into changes in cellular activity [48,49,50,51]. Through these mechanisms, Cys directly influences signaling pathways involved in neuronal adaptation and stress responses.

A major physiological role of SCAA in the brain is the maintenance of redox homeostasis. ROS are continuously generated during oxidative metabolism, mitochondrial respiration, neurotransmission and inflammatory responses [52,53]. To counteract ROS generation, neural cells rely heavily on GSH. As we mentioned before, GSH synthesis depends on Cys bioavailability and on the activity of gamma-glutamyl-cysteinyl-ligase (GCLc), the rate-limiting enzyme of the pathway [7]. In addition to serving as the precursor of GSH, free -SH groups in Cys-containing molecules can directly scavenge reactive radicals and chelate transitional metals. Depending on concentration, subcellular localization, and the surrounding redox environment, these interactions may be protective or may contribute to oxidative damage when thiol becomes dysregulated [54,55,56,57,58].

In addition, products of the TSP exert important biological effects in the nervous system. H_2_S, generated mainly by CBS, CTH and related sulfur enzymes, is now recognized as an endogenous signaling molecule with pleiotropic actions in the brain. H_2_S is produced in both the cytosol and the mitochondria and, because of its physicochemical properties, can readily diffuse across biological membranes [19]. Under physiological conditions, tissue H_2_S is maintained within a low nanomolar range and displays rapid turnover, allowing spatial and temporal regulation of its signaling activity [59]. At these concentrations, H_2_S acts as a modulator of mitochondrial function. Depending on cellular context and local concentration, it can either stimulate or restrain electron transport chain activity, thereby influencing bioenergetic flux [60]. H_2_S has also been implicated in the regulation of mitochondrial dynamics, ultrastructure and respiratory supercomplex stability. In addition, H_2_S participates in the control of antioxidant defenses, inflammatory pathways and proteostasis [19,61]. Other sulfur metabolites such as lanthionine ketimine and related compounds have shown antioxidant, neurotrophic and anti-inflammatory properties in experimental models of cerebral ischemia, neuroinflammation and neurodegeneration [18,62]. Cysteine disulfide, also known as thiocysteine (CySSH), is another TSP product that has the ability to scavenge ROS and chelate metals such as iron, copper and toxic metals [8]. This evidence suggests that non-canonical sulfur metabolites may have underappreciated physiological relevance.

Met metabolism is equally essential since it sustains cellular methylation capacity through SAM. SAM-dependent reactions are required for DNA and histone methylation, RNA processing, phospholipid synthesis and metabolism of biogenic amines [16]. During neurodevelopment, these processes are central to cell fate determination, neural differentiation, myelination, and long-term regulation of gene expression. In mature neural tissue, methylation reactions remain necessary for membrane turnover, myelin lipids such as phosphatidylcholine and sphingomyelin, and the synthesis of metabolism of catecholaminergic neurotransmitters [16]. Accordingly, disturbances in Met may impair both development and adult brain function.

Several SCAA and sulfur-derived metabolites also display neuromodulatory activity. In this context, it is important to consider that glutamatergic transmission accounts for approximately 90% of excitatory synaptic signaling in the brain. Among glutamate receptors, the NMDA receptors are essential for neurodevelopment, learning, synaptic plasticity, and memory formation and consolidation [63]. It has been described that Hcy can interact with NMDA receptors. Excessive accumulation of Hcy due to the altered flux between Met cycle and TSP has been associated with excitotoxic signaling, oxidative stress, endothelial dysfunction and increased vulnerability to neuronal damage [64]. In contrast, H_2_S can facilitate synaptic plasticity under physiological conditions by modulating NMDA receptor responses and influencing glutamate handling in neuronal cells [65]. Experimental studies further suggest that reduced content of H_2_S may contribute to stress-related behavioral phenotypes, whereas exogenous administration of H_2_S donors can improve hippocampal synaptic plasticity [17,19,66,67].

Taurine, a highly abundant sulfur-containing metabolite derived from Cys catabolism, is another key regulator of brain physiology. Although not incorporated into proteins, taurine contributes to osmoregulation, calcium modulation in neurons, retinal formation, neuronal migration and synapse formation [27]. Taurine also exerts antioxidant and cytoprotective actions, particularly during development and under conditions of metabolic stress [27].

Finally, proper SCAA transport is *per se* an important determinant of neurometabolic balance. Transporter systems can influence both intracellular Cys availability and extracellular glutamate dynamics, as in the case of Xc- system (SLC7A11), which exchanges extracellular cystine for intracellular glutamate. Consequently, altered transporter functions may simultaneously compromise redox and glutamatergic homeostasis in the brain [68,69].

Taken together, these observations indicate that SCAA metabolism is deeply integrated into brain physiology. Rather than acting as isolated metabolites, SCAAs constitute an interconnected metabolic network that integrates one-carbon metabolism, antioxidant defense, sulfur signaling, and neurotransmission. In the CNS, this network contributes to neuronal resilience by coordinating cellular response to metabolic and environmental stressors.

Given the central role of SCAA metabolism in several pathways, disruption of the dynamics of this homeostasis by environmental toxicants may have important consequences for brain function. Among these toxicants, metals are particularly interesting because they directly interact with sulfur-containing metabolites.

## 3. Metal-Induced Neurotoxicity and Potential Contribution of SCAA Dysregulation

Because many sulfur-containing metabolites possess reactive thiol groups and metal-binding properties, SCAA homeostasis is particularly sensitive to environmental toxicants. Emerging evidence suggests that disruption of sulfur metabolism may represent a common mechanism linking metal exposure with impaired neuronal resilience and increased susceptibility to neurological dysfunction [70,71,72,73,74,75].

### 3.1. Convergent Mechanisms of Metal-Induced Neurotoxicity

Metals are elements characterized by high thermal and electrical conductivity, and diverse redox properties. Although several metals are essential for biological functions at trace concentrations, others can exert toxic effects at relatively low exposure levels. Among the most relevant neurotoxic elements are lead (Pb), mercury (Hg), and cadmium (Cd), as well as the metalloid arsenic (As), all of which have been associated with adverse neurological outcomes in both experimental and epidemiological studies [12,13,76,77]. Metal deposition in soil, air and underground water is a result of natural processes. Volcanic activity, erosion, and geochemical mobilization contribute to baseline environmental metal levels, whereas mining, smelting, fossil combustion, battery production, electronic waste, industrial emissions and agricultural activities have substantially increased human exposure during the last century. In the particular case of As, the overexploitation of groundwater has increased the mobilization of naturally occurring inorganic As into drinking water reservoirs in several regions worldwide [78]. Cd exposure is strongly linked to mining and metal refining processes, while Pb and Hg contamination remain important public health concerns because of their persistence and bioaccumulation in ecosystems [79]. Human exposure occurs primarily through ingestion of contaminated water or food, inhalation of polluted air or occupational dust, and, in some settings, dermal contact. Once absorbed, many toxic metals can accumulate in tissues for prolonged periods and induce multiorgan toxicity. Importantly, the nervous system is especially vulnerable since several metals can cross the BBB, disrupting neuronal homeostasis. Although each metal exhibits distinct properties, several convergent mechanisms have been identified, including oxidative stress, mitochondrial dysfunction, impaired proteostasis, neuroinflammation, excitotoxicity and disruption of neurotransmitters systems.

Chronic metal exposure has been associated with cognitive impairment, behavioral alterations, motor dysfunction, and increased susceptibility to neurodevelopmental and neurodegenerative disorders [80,81,82]. Oxidative stress is considered one of the central mechanisms underlying metal-induced neurotoxicity. Metals such as Cd, Hg, Pb, and the metalloid As can promote excessive production of ROS, impair mitochondrial respiration, disrupt antioxidant enzymes, and deplete GSH pools [11]. Some metals participate directly in redox cycling reactions, whereas others indirectly induce oxidative damage through mitochondrial dysfunction, calcium homeostasis, inflammation, or interference with thiol-containing proteins [11]. Since SCAAs are major determinants of cellular redox buffering, disturbances in Cys availability and GSH metabolism may critically influence cellular response to metal exposure.

In addition to oxidative damage, chronic metal exposure affects neurotransmitter systems and synaptic communication. Alterations in glutamatergic, cholinergic, and dopaminergic signaling have also been observed after chronic exposure to Pb, As, and Cd in rodent studies [83,84,85]. Particularly relevant is the disruption of glutamatergic synaptic transmission through changes in NMDA receptor subunits. Experimental studies have shown altered expressions of the NR2A and NR2B receptor subunits in the cortex and hippocampus of rodents exposed to As and Pb, and these changes are correlated with impaired synaptic plasticity and cognitive deficits [86,87]. Given that glutamatergic transmission accounts for the majority of excitatory synaptic activity in the brain, alterations in glutamate homeostasis may have consequences for network function.

Mitochondrial dysfunction also represents a recurrent feature of metal neurotoxicity. Toxic metals can alter mitochondrial membrane potential, inhibit respiratory complexes, impair ATP production and trigger apoptotic signaling pathways. Considering that mitochondria are the major sources and targets of ROS, these effects frequently amplify oxidative stress and compromise neuronal survival [20]. Notably, GSH and H_2_S participate in mitochondrial protection and bioenergetic regulation, suggesting that disruption of SCAA metabolism may contribute to mitochondrial vulnerability during metal exposure. An additional mechanism involves the high affinity of many metals for sulfur-containing groups. Cys residues in proteins, GSH, and related thiol-containing metabolites are major targets of Hg, Cd, Pb, and As [11]. Binding of metals to -SH groups may alter protein folding, enzyme activity, transporter function, and redox-sensitive signaling pathways. Although these alterations can initially serve detoxification purposes by sequestering metals, sustained exposure may progressively deplete intracellular thiol pools and impair antioxidant capacity.

Interestingly, several pathological features commonly associated with neurological diseases such as PD, ASD, and AD have also been observed after metal exposure. Increased metal accumulation has been detected in patients with neurodegenerative disorders, while epidemiological studies suggest associations between environmental exposure and disease risk or progression [12,13,14,88,89]. Although causality remains difficult to establish, these observations support the hypothesis that chronic exposure to metals may act as a contributing environmental factor in susceptible individuals.

Considering the close relationship between SCAA metabolism, antioxidant defense, mitochondrial homeostasis and brain function, disruption of sulfur amino acid pathways may represent an important mechanism linking metal exposure and neurological dysfunction. Therefore, understanding how metals alter sulfur metabolic networks may provide insight into the mechanism underlying environmentally induced neurological dysfunction.

### 3.2. Toxic Metals Disrupt Sulfur Amino Acid Homeostasis

Although different metals display chemical properties and toxicokinetics, accumulating evidence indicates that many converge on common sulfur-dependent pathways as shown in Table 1 and Table 2. Studies included in this section were identified using a structured literature search strategy provided in Supplementary Materials.

#### 3.2.1. Cadmium (Cd)

Cd exposure has also been associated with altered sulfur metabolism. Epidemiological studies report increased circulating levels of Hcy in Cd-exposed populations [90] (Table 1), while experimental metabolomic analysis reveal broad disturbances in Met cycle and transsulfuration intermediates (Table 2). In rats exposed to Cd, sulfur metabolism is redirected toward increased Met remethylation rather than transsulfuration, resulting in reduced GSH levels [91]. Other studies suggest that Cd may simultaneously impair remethylation pathways by interfering with folate uptake and reducing expression of MAT2A and CBS enzymes [92,93]. Cd has high affinity for the sulfhydryl group; depletion of the intracellular thiol pool may exacerbate oxidative stress and mitochondrial dysfunction. Thus, impaired sulfur group flux through both transsulfuration or remethylation pathways could explain the combined accumulation of Hcy and reduction in GSH observed during chronic Cd exposure.

**Table 1:** SCAA metabolism in human populations chronically exposed to metals and metalloids.

Population	Exposure Levels	Main Findings	References
Cadmium			
China			
Smoking (*n* = 61) Non-smoking adult (*n* = 98)	Blood median (IQR) Male: 1.11 (0.55–2.75) µg/dL, and female: 0.72 (0.29–1.33) µg/dL	Blood Cd levels are positively associated with blood Hcy. Higher Hcy levels in the smoking vs. non-smoking population	[90]
Arsenic (As)			
Argentina			
Lung cancer patient (*n* = 109) Control (n = 141)	Water < 200 µg/L	Lung cancer risk increased with the increase in MMA levels (%) in urine. The risk is associated with CBS SNP.	[94]
Brazil			
35–50 years old Taxi drivers (*n* = 42) Non-smoking men controls (*n* = 27)	Blood levels Drivers: 14.87 ± 1.01 µg/L Control: 9.26 ± 0.96 µg/L	As levels are positively associated with Hcy and proinflammatory cytokines (IL-1β, IL-6, TNF-α) and negatively associated with GPx activity, in taxi drivers	[95]
Mexico			
Pregnant women (*n* = 197)	Water: 24.7 (0.33–235.6) µg/L	Vitamin B12 deficiency in the population. Maternal vitamin B9/B12 levels positively correlate with urine MMAs, and DMAs in cord serum.	[96] *
India			
18–86 years old Populations with access to drinking water with low As vs. high As levels Control (*n* = 193) Exposed (*n* = 226)	Water: Control < 10 µg/L As exposure > 50 µg/L Urinay Control male: 5 ± 5.15 µg/L, Control female: 5.2 ± 5.9 µg/L As-exposed male: 65.4 ± 82.3 µg/L As-exposed female: 51.7 ± 51.1 µg/L	Vitamin B9/B12 deficiency increased with As consumption. As decreased Cys and increased Hcy plasmatic levels	[97] *
Bangladesh			
20–65 years old (*n* = 1650) 6-year-old children (*n* = 165) 30–65 years old (*n* = 353)	Water: <650 µg/L Urine-As Female: 97.6 ± 119.7 µg/L Male: 121.4 ± 140.4 µg/L Median (Range) Water: 114 (0–700) µg/L Urine: 124 (3–1990) µg/L Blood: 10.8 (1.2–57.0) µg/L	Higher prevalence of hyperhomocysteinemia. As levels in water are negatively associated with plasma folate. Urine DMA is positively associated with folate levels and negatively associated with Hcy. Marginal folate levels in 20% of children. Urine iAs (%) inversely correlated with blood folate and Cys levels. Blood SAM levels are negatively associated with urinary As. Vitamin B9/B12 status modified this association, no association with SAH	[98] * [99] * [100] * [101] *
Taiwan			
>40 years old Patient with carotid atherosclerosis (*n* = 163) Control (*n* = 163)	Water < 3590 µg/L	Cumulative As exposure positively correlated with blood Hcy levels. High Hcy and MMA% levels increased the risk of developing atherosclerosis 5.4-fold.	[102] *
Lead (Pb)			
USA			
>20 years old (*n* = 4089) >20 years old (*n* = 4482) 20–59 years old (*n* = 2492) men > 55 years old (*n* = 1218) New York, women 18–44 years old (*n* = 259) Maryland older adults 50–70 years old (*n* = 1037)	Blood Mean ± SD (Range): 2.1 ± 1.8 (0.2–33.0) µg/dL Blood Mean ± SEM: 1.75 ± 0.04 μg/dL Blood Mean ± SEM: 2.88 ± 0.13 µg/dL Blood Mean ± SD: 4.9 ± 2.7 μg/dL Blood Mean (95% CI): 0.91 (0.86–0.96) μg/dL Blood Mean ± SD: 3.5 ± 2.4 µg/dL	Blood Pb levels positively correlated with blood Hcy in vitamin B6, B9, B12-deficient population. Increased hyperhomocysteinemia prevalence. Pb levels were strongly correlated with blood Hcy, C reactive protein, cholesterol, and BMI in patients with cardiovascular disease.	[103,104,105,106,107] * [108]
Pakistan			
Low-income population 18–60 years old (*n* = 872)	Blood median (IQR): 10.82 µg/dL (8.29–13.60)	Subjects in the higher Pb levels quartile presented higher levels of Hcy. Model was adjusted for age, gender, folate and vitamin B12.	[109] *
Poland			
Occupationally exposed men (20–60 years old) (*n* = 231) Metal workers (25–55 years old) (*n* = 183)	Blood Median (95% CI): 2.56 (0.9–4.72) μg/dL Blood Range Low Exposure: 20–45 μg/dL High exposure: 45–60 μg/dL	High Pb levels positively correlated with Hcy levels, C reactive protein, fibrinogen. Workers with higher Pb levels had higher blood Hcy, high carbonylated proteins, and lower blood GSH levels.	[110,111]
South Korea			
41–71 years old (*n* = 386)	Blood Mean ± SD: 4.4 ±1.9 µg/dL	Blood Pb levels positively correlated with plasma Hcy. Polymorphism in CBS, MTR and MTHFR did not show interaction with blood levels.	[112]
Ukraine			
Occupationally exposed men (38–47 years old) (*n* = 146) Control (*n* = 57)	Blood Mean ± SEM: High exposure: 2.12 ± 0.56 μmol/L Low exposure: 1.72 ± 0.03 μmol/L	Higher Pb and Hcy levels in patients with cardiovascular manifestations. Positive correlation between Hcy and Pb levels.	[113]
Singapore and Vietnam			
Battery factory workers (19–66 years old) Singapore (*n* = 183) Vietnam (*n* = 323)	Blood Mean (Range): 22.7 (2.0–66.9) µg/dL	Positive correlation between blood Pb and plasma Hcy.	[114]
Brazil			
35–50 years old Taxi drivers (*n* = 42), Non-smoking men controls (*n* = 27) Male adult (*n* = 45)	Blood Mean ± SEM: Drivers: 2.60 ± 0.21 µg/dL, Control: 1.95 ± 0.24 µg/dL Blood Mean ± SEM: 11.38 ± 1.92 µg/dL	Pb levels are positively associated with Hcy and proinflammatory cytokine levels (IL-1β, IL-6, RNF-α). Subjects with blood Pb > 5 µg/dL had lower plasmatic H_2_S levels.	[66,95]
China			
Smoking (*n* = 61), Non-smoking adult (*n* = 98)	Blood Median (IQR) male: 35.91 (23.45–48.77) μg/dL female: 27.10 (21.46–32.76) μg/dL	Hcy levels correlated with Pb levels, especially in smoking men.	[90]
India			
Pb-exposed workers male > 18 years old (*n* = 338)	Blood Range <10 µg/dL, 10–30 µg/dL, 30–50 µg/dL, >50 µg/dL	Blood Pb levels > 10 µg/dL decreased SAM levels and methylation index, while SAH levels increased. Lifestyle, working experience > 5 years and age, synergically influence in SAM levels.	[115]
Aluminum			
Italy			
18–75 years old	Urine >35 μg/g creatinine	Hyperhomocysteinemia. Chelating therapy reduced serum Hcy and ROS levels and increased serum vitamins B9 and B12, increased GSH, antioxidant capacity, and Al elimination.	[116] *
Mercury			
Brazil			
35–50 years old Taxi drivers (n = 42), Non-smoking men controls (*n* = 27).	Blood Mean ± SEM Drivers: 33.65 ± 2.95 µg/L, Control: 11.8 ± 1.57 µg/L	Increased Hg blood levels in drivers. Hg levels are positively associated with Hcy levels and negatively with GPx activity. Increased IL-1β, IL-6, TNF-α in taxi drivers.	[95]
USA			
NHANES study Children 3–5 years (*n* = 1005)	Blood Q1: ≤0.70 μmol/L, Q2: 0.70–1.50 μmol/L, Q3: 1.50–3.49 μmol/L, Q4: >3.49 μmol/L	Inverse association between Hg and Hcy in boys, but not in girls with lower vitamin B9/B12.	[117] *
Oman			
Autistic children (*n* = 27) Control (*n* = 27)	Hair (mean ± SD) Autistic: 6.93 ± 0.36 mg/g, Control: 0.611 ± 0.033 mg/g	Vit B9/B12 deficiency in children. Lower serum GSH levels and higher Hcy and SAH levels in autistic children. Hair Hg levels were markedly elevated in autistic subjects and inversely associated with Cys levels.	[70] *

* Research studies that consider nutritional status, especially levels of Vitamin B6, B9 and B12. Hyperhomocysteinemia (children–adolescent) > 7 µmol/L, Hyperhomocysteinemia (adult) > 15 µmol/L, B9 Deficiency < 3 ng/mL or 6 nmol/L, B12 Deficiency < 200-pg/mL or 150 pmol/L, B6 Deficiency < 5 µg/L or 20 nmol/L. SEM: standard error of the mean, SD: standard deviation, IQR: interquartile range, 95% CI: 95 confidence intervals, BMI: body mass index, SNP: single nucleotide polymorphism. MMA: monomethylated arsenic, DMA: dimethylated arsenic, GPx: glutathione peroxidase, Cd: cadmium, As: arsenic, Pb: lead, Al: aluminum, Hg: mercury, SAH: S-adenosylhomocysteine, Hcy: homocysteine, Cys: cysteine, H_2_S: hydrogen sulfide.

#### 3.2.2. Arsenic (As)

Among environmentally relevant toxicants, As is biotransformed inside the cell. As is metabolized by repetitive oxidative methylation reactions catalyzed by As3+ methyltransferase (As3MT). These reactions required SAM as a methyl donor and reducing equivalents supplied mainly by GSH or thioredoxin systems. Consequently, As metabolism imposes a substantial metabolic demand on one-carbon and sulfur pathways, potentially leading to depletion of both SAM and GSH pools [118]. Variability in As toxicity and As metabolites profiles among exposed individuals has been associated with nutritional status, particularly folate, and B complex vitamin and Met intake, as well as with polymorphisms in genes involved in sulfur metabolism, including MTHRF, MTR and CBS [118,119,120,121,122,123,124,125,126]. Epidemiological studies further report elevated Hcy and reduced circulating folate or cobalamin levels in As-exposed populations, suggesting impaired remethylation capacity (Table 1) [97,99,102,127,128]. Experimental studies support this interpretation (Table 2). Reduced MTR activity has been reported in the SH-SY5Y cells exposed to As, apparently through redox-dependent mechanisms affecting enzyme function [129,130]. Similarly, reduced expression of BHMT has been shown in liver tissue from gestationally exposed mice and in human prostate cell lines [131,132,133]. These alterations may redirect Hcy metabolism toward transsulfuration in an attempt to increase Cys availability for GSH synthesis and metal detoxification. Nevertheless, persistent pathway dysregulation can also drive higher Hcy accumulation and compromise redox balance.

Consistent with this complexity, As exposure has been associated with both activation and impairment of transsulfuration depending on exposure conditions and tissue context. Increased CBS expression and enhanced transsulfuration activity have been reported in liver and brain cortex after As exposure [131,133,134,135], possibly reflecting an adaptive response. In contrast, chronic exposure to Realgar, an arsenic-containing mineral, reduced hippocampal H_2_S, Cys, and GSH levels in mice, changes accompanied by memory impairment and oxidative stress [72]. Similarly, combined gestational exposure to As and fluoride decreased transsulfuration enzyme activity in the cortex and hippocampus of offspring [86].

**Table 2:** SCAA alterations in experimental models of exposure to metals and metalloids.

Model	Exposure Dose	Main Finding	References
Cadmium (Cd)			
Chang cell line	CdCl_2_ 1 µM for 1 h + fresh medium for up to 24 h	↑ CTH protein and H_2_S production in a time-dependent manner, inducing an adaptative response to radiation.	[136]
C2C12 cell line (Murine Myoblast)	CdCl_2_: 1–100 µM (30 µM) for 24 h	↑ CTH expression and H_2_S production, inhibition of CTH aggravate Cd-induced apoptosis.	[137]
C57BL/6J mice and CTH KO (C57BL/6J background)	CdCl_2_ 5 mg/kg (i.p) for 24 h	RSS produced by CTH neutralized Cd through the formation of CdS. CTH deletion enhanced Cd-induced hepatotoxicity.	[138]
Male Sprague-Dawley rats	CdCl_2_ 0.6 mg/kg/day (s.c), 5 day/week for 6 weeks	↓ SAM, cystathionine, MAT2B and CBS proteins in the kidney, associated with nephrotoxicity and oxidative stress (↑ MDA, 4HNE and ↓ GSH).	[92]
Arsenic (As)			
RWPE1 human non-tumorigenic prostate cell line (do not readily methylate As)	NaAsO_2_ 5 µM up to 16 weeks	↑ MAT2B, SAHH, CBS, GSH-synthesis enzymes, and MRP1 transcript in a time-dependent manner. ↓ MAT2A transcript but returned to normal levels by 12 weeks. ↓ BHMT transcript.	[131]
SH-S5Y5 cells	NaAsO_2_ Up to 10 µM	↓ GSH and Cys levels and inhibited MTR activity.	[130]
N2a cell line (neuron) + N9 cell line (microglia)	NaAsO_2_ 10 and 20 µM	As induces extracellular Cys depletion by N9 cells, dependent on SLC7A11 overexpression in N9 cell, leading to neuron oxidative cell death.	[71]
HepG2 hepatocellular carcinoma cell line Macaca fascicularis	NaAsO_2_ 3.75, 7.5, 15 µM for 24 h NaAsO_2_ 1 mg/kg/day for 28 days	↓ CBS and GPx1 protein in cell line and ↓ monkey liver.	[139]
CH3 mice	NaAsO_2_ 85 mg/L from embryonic day 8–18 (model of hepatocellular carcinoma)	In fetal liver: ↓ MAT1A and BHMT expression. In newborn liver: ↑ GSH-synthesis genes and metallothionein expression and ↓ BHMT expression	[132] [133]
Male ICR mice	Realgar 0.15, 0.45, 1.35 g/kg/day (i.g) for 8 weeks	↓ GSH, RSS, cysteine, and cystine transporters in the hippocampus. Alteration associated with ultrastructural changes and cognitive deficiencies (NOR test).	[72]
C57BL/6 mice	NaAsO_2_ 5–10 mg/L for 6 months	↓ SAM levels, MTR expression and activity in liver, and vitamin B9 and B12 (especially at 5 mg/L) in serum, associated with lipid accumulation and inflammation in liver, and microbiota dysbiosis (↑ vitamin B9/12 producing bacteria). Serum: ↓ Met, SAM levels, ↑ SAH Liver: ↓ SAM associated with ↓ m6A RNA modification, inducing ↑ fatty acid synthesis and NAFLD phenotype.	[140,141]
CD1 mice	NaAsO_2_ 20 mg/L from gestation until 3 months after birth NaAsO_2_: 2 mg/L + NaF: 25 mg/L (oral) from gestation until 3 months after birth	↑ GSH levels in cortex and hippocampus, associated with ↑ SLC1A1, SLC7A5 and SLC7A11 protein, and alteration in glutamate receptor and cognitive impairment (LOR test) As and As+F ↓ the activity of the transsulfuration pathway in the cortex and hippocampus at 1 month and 3 months after birth, associated with cognitive impairment (NOR/LOR test).	[73,86]
Female Wistar rat	NaAsO_2_ 3 mg/L from gestation up to 4 months old	↓ SAM and phosphatidylcholine levels and ↑ choline levels in the liver, associated with histological alteration in liver. No changes in the brain, although important myelin and nerve alteration in the striatum were observed.	[142]
Male Sprague Dawley rat	NaAsO_2_ 5, 10, 50 mg/L for 6 months	As (10 and 50 ppm) ↑ Hcy levels in the brain and serum and induced histopathological changes in CA1, endoplasmic reticulum stress and memory impairment (MWM test).	[143]
Lead (Pb)			
SH-S5Y5 cell line	Pb(NO_3_)_2_ 100 nM for 60 min	↓ GSH levels and Cys uptake and inhibited MTR activity.	[130]
C57BL/6J mice	PbAc 30 mg/L 100 mg/kg (oral) from P21–P60 Followed by alcohol seeking paradigm.	Pb ↑ propensity to alcohol seeking relapses, associated with ↑ SLC7A11 expression in nucleus accumbens and dorso-lateral striatum, and aberrant glutamatergic transmission	[74]
Rat	PbAc 120 mg/kg (oral) for 60 days	↓ Cys and Met levels in the liver.	[144]
Fisher 344 rats	PbAc 2000 mg/L for 5 weeks	↓ Cys and GSH, and ↑ MDA levels in rat lenses	[145]
Male Wistar rat	PbAc 100 mg/kg (oral) for 1 and 3 months	↓ H_2_S level, CTH expression, and GSH levels in the kidney. ↑ oxidative stress, inflammation, and ↓ H_2_S and CTH protein levels in the liver. NaHS treatment recovered GSH levels, ↑ CTH expression, ↓ liver damage and inflammation, and reduced renal toxicity.	[146,147]
Aluminum (Al)			
SH-SY5Y cell line	Up to 10 µM	↓ GSH levels and Cys uptake and inhibited MTR activity.	[130]
Male Wistar rat	AlCl_3_ 50 mg/kg/day (oral) for 3 months	↑ blood Hcy levels, AchE, BACE1, and IL1β levels in the brain. Induced cognitive impairment (MWM test).	[148]
Male Wistar rat (Al-induced AD model)	AlCl_3_ 17 mg/kg/day (oral) for 4 weeks	↓ antioxidant capacity, acetylcholine, and dopamine levels, ↑ Hcy levels, induced histological degeneration in the cortex and cerebellum, and behavioral impairment (OFT).	[149]
Mercury			
HeLa cell line	HgCl_2_ 1–100 µM for 24 h	At 10 µM: ↑ GSH levels, Cys and Hcy utilization, and GSSG release. At 100 µM: ↓ GSH levels and delayed cell growth.	[150,151]
Cerebellar granular neuron, Cell line: PC12, NPC1231, SH-S5Y5	MeHg 2 µM for 24 h	↓ RSS levels and activated redox signaling. RSS pretreatment attenuated cell damage and oxidative stress	
SH-S5Y5 cell line	TH: 10 µM HgCl_2_: 10 µM NaHS (500 µM) for 12 h +/− MeHg (0.5–2 µM) for 24 h	↓ MTR activity by a redox-induced GSCbI/MeCbl cofactor imbalance and indirectly through IGF1 and dopamine-mediated signaling. Pretreatment with NaHS or CBS overexpression suppressed MeHg toxicity. Endogenous RSS interacts in vitro with MeHg to form an inert metabolite (MeHg)_2_S	[129,130]
ICR mice	HgCl_2_ 1.5, 3, and 4.5 mg/kg/day from embryonic day 5.5–9.5	Hg alters SCAA flux between placenta and embryos. In placenta: ↑ oxidative stress, SLC7A5 and SLC7A11 expression. In embryos: ↑ Cys and Met, but ↓ SAM and Hcy SCAA metabolism imbalance in embryo is associated with neural tube defect.	[152]
C57BL/6J mice and CTH KO (C57BL/6J background)	MeHg 5 mg/kg/day for 12 days (oral)	CTH KO mice: ↑ MeHg levels in the cerebellum, and higher susceptibility to MeHg-induced motor impairment (rotarod test) and cerebellum damage	[75]
Rat	HgCl_2_ 1 mg/kg/day for 45 days (s.c)	↑ Cys levels in a time-dependent manner. ↑ Met levels increased after 15 days but returned to control levels on the 45th day.	[144]
Small Indian mongoose (Herpestes auropunctatus)	Natural exposure Liver MeHg levels Mean (range): 12.7 (1.75–55.5) µg/g	Mongoose expresses CBS/CTH in different tissues and produces significant RSS levels, including CySSH, H_2_S, and (MeHg)_2_S metabolites.	[153]

Cd: cadmium, As: arsenic, Pb: lead, Al: aluminum, Hg: mercury, MeHg: methylmercury, Met: methionine, SAM: S-adenosylmethionine, Hcy: homocysteine, Cys: cysteine, H_2_S: hydrogen sulfide, GSH: glutathione, NaHS: sodium hydrosulfide, CySSH: thiocysteine, RSS: sulfur reactive species, MAT2A: methionine adenosyltransferase subunit 2A, MAT2B: methionine adenosyltransferase subunit 2A, MTR: methionine synthase, BHMT: betaine–homocysteine methyltransferase, CBS: cystathionine β synthase, CTH: cystathionine γ lyase, MRP1: multidrug resistant-associated protein 1, SLC1A1: solute carrier family 1 member 1 (also known as EACC1 or EAAT3), SLC7A11: solute carrier family 7 member 11 (also known as xCT), GSCb: glutathionyl cobalamin, MeCbl: methyl cobalamin, IGF1: insulin growth factor 1, GPx1: glutathione peroxidase 1, AchE: Acetylcholine esterase, BACE1: beta-site APP cleaving enzyme 1, IL1β: interleukine 1 beta, KO: knock out. NOR: Novel Object Recognition, LOR: Location Object Recognition, MWM: Morris Water Maze, OFT: Open Field Test. ↑ and ↓ arrows indicate increase and decrease, respectively.

#### 3.2.3. Lead (Pb)

Pb exposure has long been associated with cognitive impairment and altered neurodevelopment, particularly during early life [81,154]. Several human studies report positive associations between circulating Pb levels and blood Hcy levels and reduced levels of the vitamins involved in its metabolism, obtaining a stronger association between Pb and Hcy concentrations, especially in populations with low dietary intake of folate and vitamins B6/B12. Reduced levels of plasmatic H_2_S have also been reported in Pb-exposed subjects, suggesting broader disruption of sulfur metabolism (Table 1). Experimental models support these observations. Rodents exposed to Pb (100 mg/kg/day) showed altered expression and activity of the Met cycle and transsulfuration enzymes, such as MTR and CTH (Table 2). Because Pb can interfere with redox-sensitive proteins and calcium-dependent signaling pathways, these metabolic disturbances may contribute to impaired glutamatergic transmission, oxidative stress and synaptic dysfunction frequently observed after Pb exposure.

#### 3.2.4. Aluminum (Al)

Chronic exposure to Al has been associated with oxidative stress and neurodegenerative-like alterations in experimental models and selected human studies. Elevated Hcy concentrations and depletion of antioxidant defenses have been reported in both Al-exposed animals and humans, suggesting dysregulation of SCAA metabolism [116,148] (Table 1 and Table 2). In rodent models, Hcy accumulation following Al exposure showed an adverse effect on the brain and was associated with histological degeneration, inflammation, and cognitive and locomotor impairment [149,155]. Although the mechanisms remain incompletely understood, disruption of one-carbon metabolism and impaired antioxidants are likely contributors to these effects.

#### 3.2.5. Mercury (Hg)

Hg, particularly in its methylmercury form (MeHg), displays high affinity for sulfur-containing molecules. Hg readily forms complexes with Cys, GSH and protein thiols, affecting SCAA and redox signaling. Epidemiological studies have associated Hg exposure with higher levels of Hcy and reduced folate levels in the blood [95,156]. In children, higher Hg concentrations in blood have also been linked to alterations in psychomotor development scores, especially under conditions of low folate availability [157]. Alterations in SCAA are evident in studies involving ASD. Children with ASD from Oman (regions with elevated Hg exposure) presented higher levels of Hg in hair and reduced serum GSH together with increased Hcy, SAH, and N-homocysteinylation, consistent with oxidative stress and impaired methylation capacity [70]. Interestingly, an earlier study from the same population identified deficiencies in folate, and Vitamin B9 and B12 intake, suggesting that nutritional status may influence susceptibility to Hg toxicity [70] (Table 1). Mechanistically, Hg can inhibit MTR, CBS, and CTH activity in vitro, which could affect the Met remethylation and transsulfuration pathway. However, these results should be carefully considered because the concentration used may not resemble tissue physiological levels [129,158,159,160]. Contrastingly, in vivo studies show the induction of CTH and/or CBS and reactive sulfur species (RSS) after Hg exposure, indicating adaptive detoxification responses (Table 2). Sulfur-containing metabolites, including Hcy-MeHg and GSH-MeHg complexes, facilitate Hg sequestration and elimination through multidrug resistance-associated proteins (MRPs) [161]. Formation of sulfur-rich metabolites such as bis(methylmercury) sulfide ((MeHg)_2_S) has also been detected in exposed rodents and appears highly dependent on CTH activity [75,162,163].

**Figure 4:**
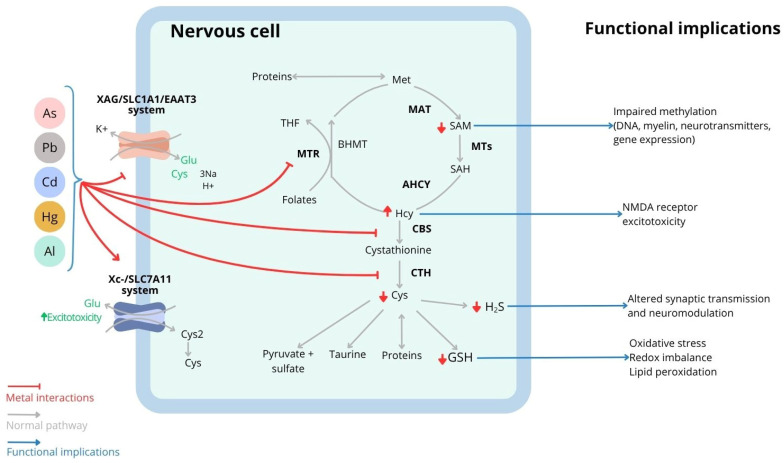
Heavy metal disruption of sulfur-containing amino acid (SCAA) homeostasis in nervous cells. This schematic emphasizes the mechanistic link between heavy metal exposure and neurotoxic outcomes. Arsenic (As), lead (Pb), cadmium (Cd), mercury (Hg), and aluminum (Al) interfere with SCAA homeostasis through transport systems (XAG/SLC1A1/EAAT3 and Xc-/SLC7A11), and inhibition of related enzymes. Red arrows indicate toxic metal interactions, gray arrows depict physiological flux, and blue arrows highlight functional consequences. Disruptions result in impaired methylation (DNA, myelin, neurotransmitters, gene expression), NMDA receptor excitotoxicity, altered synaptic transmission, and oxidative stress with redox imbalance and lipid peroxidation. Metabolites: tetrahydrofolate (THF), methionine (Met), cysteine (Cys), homocysteine (Hcy), S-adenosylmethionine (SAM), S-adenosylhomocysteine (SAH), glutathione (GSH), taurine, and hydrogen sulfide (H_2_S). Enzymes: methionine synthase (MTR), betaine–homocysteine methyltransferase (BHM)T, methionine adenosyltransferase (MAT), S-adenosylhomocysteine hydrolase (AHCY), cystathionine β synthase (CBS), cystathionine γ lyase (CTH). ↑ and ↓ red arrows indicate increase and decrease, respectively.

Collectively, these findings indicate that sulfur metabolism is not a passive target of metal toxicity but rather an active component of the adaptative response to environmental stress. Toxic metals disrupt SCAA homeostasis at several interconnected levels, including one-carbon metabolism, transsulfuration, GSH synthesis, and sulfur-dependent detoxification pathways (Table 1 and Table 2). Although each metal has distinct chemical properties, many converge on thiol-containing metabolites and redox-sensitive enzymes. Based on the evidence reviewed here, metal exposure alters SCAA metabolism through enzyme inhibition, impaired amino acid transport, and increased consumption of Cys and SAM during detoxification responses (Figure 4). This imbalance may reduce methylation capacity, promote Hcy accumulation, limit GSH and H_2_S production, and in consequence compromise neurotransmission, proteostasis, mitochondrial dysfunction and neuronal resilience. These shared alterations may help explain how chronic metal exposure increases vulnerability to neurological diseases.

## 4. Dysregulation of SCAA Metabolism in Neurological Disorders

Although neurodegenerative and neurodevelopmental disorders differ in their etiology and clinical manifestations, many share common pathological mechanisms, including oxidative stress, mitochondrial dysfunction, impaired proteostasis, neuroinflammation, and altered neurotransmission [2,3,19,164]. As discussed in the previous section, chronic exposure to toxic metals induces similar alterations while disrupting sulfur amino acid homeostasis. This overlap suggests the possibility that dysregulation of SCAA metabolism may represent a convergent mechanism linking environmental exposures to increased susceptibility to neurological diseases.

### 4.1. Alzheimer’s Disease (AD)

AD has been associated with multiple abnormalities in sulfur metabolism, including reduced GSH and H_2_S levels, decreased SLC1A1 expression, elevated Hcy levels and decreased SAM levels in the brain and peripheral compartments [164,165,166,167]. These changes are relevant if we consider that these dysregulations may simultaneously impair antioxidant defense, methylation capacity and synaptic function. In fact, hyperhomocysteinemia has been linked to cognitive decline, vascular dysfunction and increased dementia risk, whereas reduced SAM may compromise epigenetic regulation and phospholipid metabolism [167,168]. Genetic variants in enzymes involved in one carbon and transsulfuration metabolism such as MTHFR, CBS, and CTH, as well as deficiencies of folate, vitamin B6 and vitamin B12, have been considered risk factors for dementia and late-onset AD [166,168,169]. Mechanistically, a decrease in SAM availability leads to diminished transsulfuration flux, resulting in decreased levels of H_2_S and GSH. Experimental studies support this interpretation since restoration of H_2_S signaling using sodium hydrosulfide (NaHS) in APP/PS1 transgenic mice downregulates BACE1 enzyme expression, leading to an attenuation of Aβ plaques formation, reduces phosphorylation of amyloid precursor protein and tau proteins and ameliorates memory impairment [17,170].

### 4.2. Huntington’s Disease (HD)

HD results from an expanded CAG repeat in the huntingtin gene, leading to a mutant huntingtin protein (mHTT) with an elongated polyglutamine tract. In addition to its well-known effects on transcriptional regulation, proteostasis and mitochondrial function, mHTT also disrupts SCAA metabolism. Experimental evidence indicates that mHTT interferes with expression and trafficking of key Cys-related transporters, including SLC1A1 and SLC7A11, thereby affecting Cys availability and glutamatergic homeostasis [171,172]. One relevant mechanism involves sequestration of the SP1 transcription factor, which controls the expression of CTH [4]. Reduced CTH mRNA and protein levels have been observed in the brains of HD patients and experimental models [4]. Impaired expression of CTH may restrict Cys generation through transsulfuration and reduce endogenous H_2_S production. In addition, adaptive responses to Cys limitation are also affected in HD models, further compromising antioxidant resilience [173]. Also, reduced basal cysteine availability may contribute to the glutamatergic abnormalities and depressive-live phenotypes observed in HD [174]. These combined alterations are associated with the increased vulnerability to oxidative stress and glutamatergic dysfunction [3].

### 4.3. Parkinson Disease (PD)

PD is characterized by dopaminergic neurodegeneration, mitochondrial dysfunction, protein aggregation and oxidative stress. Several lines of evidence indicate that Cys and H_2_S metabolism have also been affected in the context of PD [2,3]. Parkin, an E3 ubiquitin ligase involved in mitochondrial quality control and protein turnover, can be activated by sulfhydration, a cysteine-based post-translational modification promoted by H_2_S. Reduced levels of sulfhydrated parkin have been observed in postmortem brains of PD patients, suggesting loss of this protective regulatory mechanism [175]. Also, Hcy was elevated in the serum of PD patients during DOPA treatment, where methylation-dependent drug metabolism may influence one-carbon balance [176,177]. In experimental models, overexpression of CBS or administration of H_2_S donors exerts a neuroprotective effect against 6-hydroxydopamine-induced toxicity, supporting the role for sulfur signaling in PD-related damage [178,179].

### 4.4. Multiple Sclerosis (MS)

MS is a chronic autoimmune disease, characterized by demyelination, oxidative stress, immune dysregulation and mitochondrial dysfunction. Several studies have reported increased serum levels of Hcy, and broader alterations in sulfur metabolites in MS patients [180,181,182,183]. Metabolomic analyses in the gray matter of postmortem patients showed a decreased concentration of SAM, betaine, and cystathionine relative to controls, suggesting altered remethylation dynamics and reduced activity in the TSP [184]. In addition, genetic association studies have been related variants in MTHFR, CBS, and folate carrier 1 polymorphisms with susceptibility or clinical features of relapsing-remitting MS in the German population [185]. Moreover, exposure of cultured human endothelial brain cells to proinflammatory cytokines such as TNFα and IFNγ induced downregulation of CBS and CTH expression, indicating that inflammatory signaling altered H_2_S production and sulfur metabolism in endothelial cells in the brain [186].

### 4.5. Autism Spectrum Disorder (ASD)

Increasing evidence suggests that altering one-carbon metabolism and redox imbalance may contribute to a subset of ASD cases. Polymorphisms in MTHFR and CBS and related folate-cycle genes have been observed in some pediatric cohorts and have been associated with symptom severity in selected studies, although are not fully consistent across populations [187,188,189]. Biochemical studies have described lower levels of GSH and reduced levels of sulfur metabolites including Cys, Met, cystathionine, taurine, and sulfate in children with ASD, suggesting alterations in the Met cycle and TSP [190,191]. On this basis, nutritional interventions aimed at supporting these pathways include the administration of folate, vitamin B6, vitamin B12 and betaine supplementation [40]. While responses are heterogeneous, some studies demonstrated improvements in oxidative stress markers or clinical outcomes, highlighting the potential relevance of metabolic stratification in ASD management.

Epidemiological studies further support a link between chronic metal exposure and neurological diseases. Occupational and environmental exposure to metals such as Pb, mercury, cadmium, arsenic and manganese have been associated with an increased risk of cognitive decline and neurological disorders [70,192,193,194,195]. For example, cumulative Pb exposure assessed by bone Pb levels has been associated with a higher risk of PD [196]. Likewise, chronic exposure to arsenic through contaminated drinking water has been linked to cognitive impairment and accelerated cognitive decline in exposed populations [197,198]. Prenatal and early-life exposure to toxic metals, including Pb and mercury, has also been associated with an increased risk of neurodevelopmental disorders, although the evidence for autism spectrum disorder remains heterogeneous [39,199]. Collectively these observations support the hypothesis that environmental disruption of sulfur amino acid metabolism may contribute to increased susceptibility to neurological disease in exposed individuals.

## 5. Therapeutic Implications and Future Perspectives

The evidence discussed throughout this review highlights the close relationship between SCAA homeostasis and resistance to metal toxicity. Accordingly, strategies aimed at preserving sulfur metabolic pathways have emerged as promising approaches to enhance neuronal resilience under conditions of environmental exposure. Supplementation with sulfur precursors, methyl donors, and vitamins required for one-carbon metabolism has shown protective effects in both experimental models and exposed populations [200,201,202,203,204,205,206,207,208,209,210,211,212]. Folate, Met, N-acetylcysteine and vitamins B6/B12 supplementation have been reported to attenuate oxidative stress and tissue injury induced by As, Cd, Pb and Al exposure [210,213,214,215,216,217,218,219]. Similarly, administration of H_2_S donors such as sodium hydrosulfide (NaHS) or diallyl trisulfide (DATS) mitigates neurotoxicity and oxidative damage in animals models exposed to these metals [66,67,149,163,220,221,222]. In parallel, sulfur-containing phytochemicals have attracted considerable attention as complementary strategies to counteract metal-induced toxicity. Plant-derived organosulfur compounds such as S-allyl-L-cysteine, diallyl sulfide, diallyl disulfide, and sulforaphane exert protective effects in multiple experimental models by restoring GSH synthesis, scavenging of reactive species, modulation of mitochondrial function and direct metal chelation [223,224,225,226,227]. However, the current evidence suggests that their beneficial effects are largely mediated through activation of the Nrf-2-dependent antioxidant response rather than by direct restoration of SCAA homeostasis.

Chelation therapy remains the standard treatment for severe cases of metal intoxications [116,228,229,230,231,232,233]; however, chelating agents alone may not fully restore redox balance or sulfur metabolic capacity. Consequently, combining chelation strategies with antioxidants sulfur donors or methyl donor supplementation have emerged as potential approaches to enhanced detoxification while preserving neuronal function [203,234,235,236,237,238,239]. Nevertheless, the efficacy and safety of these combined interventions require further investigation.

Overall, current evidence supports the idea that SCAA metabolism is both a target and compensatory component of cellular response to toxic metal exposure. Disruption of these pathways may therefore represent an important mechanistic intersection between environmental toxicology and neurological diseases. However, additional studies are needed to determine whether interventions aimed at restoring SCAA homeostasis can effectively reduce the long-term neurological consequences of metal exposure.

## 6. Concluding Remarks

SCAA and their derived metabolites form a highly interconnected metabolic network that supports essential aspects of brain physiology, including redox regulation, methylation capacity, neurotransmission, mitochondrial activity, and cellular adaptation to stress. Throughout this review, we highlight how sulfur metabolism integrates multiple processes involved in neuronal adaptation to environmental conditions. Evidence indicates that toxic metals disrupt SCAA homeostasis at several levels, including one-carbon metabolism, transsulfuration, GSH synthesis, and sulfur-dependent signaling pathways. These alterations overlap with metabolic signatures reported in neurodegenerative and neurodevelopmental disorders, supporting the hypothesis that disruption of sulfur metabolism may contribute to increased neurological vulnerability in exposed individuals.

An important aspect highlighted by current evidence is the dual role of sulfur metabolism during xenobiotic exposure. On one hand, sulfur-containing metabolites participate directly in detoxification through metal binding, antioxidant buffering and reactive sulfur species production. On the other hand, sustained activation of these adaptive responses may redistribute sulfur resources away from other essential pathways, potentially affecting neuronal signaling, bioenergetics and cellular resilience.

Despite the growing body of evidence from experimental models and exposed populations, most available studies remain descriptive and do not establish causal relationships between metal exposure, SCAA dysregulation, and neurological outcomes. There is a lack of longitudinal human studies integrating early-life metal exposure assessment with measurements of SCAA-related metabolites and long-term neurological follow-up. Such studies will be necessary to determine whether metal-induced disruption of sulfur metabolism precedes disease onset and contributes to the development of neurodegenerative diseases. This review also underscores several limitations in the current literature. Most available studies have focused predominantly on one-carbon metabolisms and GSH-related pathways, whereas the contribution of the TSP and reactive species of sulfur to brain physiology and toxicology remains comparatively underexplored. Another relevant consideration is the potential influence of genetic and nutritional variability on susceptibility to neurological diseases and metal toxicity.

Overall, the available evidence suggests that SCAA metabolism occupies a strategic position at the intersection of environmental toxicology, redox biology and brain disease. Although our proposed framework requires validation in prospective human studies, it provides a mechanistic basis for understanding how chronic metal exposure may reduce neuronal resilience and increase susceptibility to neurological disease.

## Data Availability

No new data were created or analyzed in this study.

## Supplementary Materials

The following supporting information can be downloaded at: https://www.mdpi.com/article/10.3390/metabo16070461/s1, Supplementary Material: Section S1.2.2 Genes involved in SCAA homeostasis are differentially expressed across the brain, Section S3.2 Toxic metals disrupt sulfur amino acid homeostasis.

## Abbreviations

The following abbreviations are used in this manuscript:

AD	Alzheimer Disease
AHCY	S-adenosylhomocysteine hydrolase, also SAHH
Al	Aluminum
APP/PS1 mice	Doble transgenic strain with mutations in amyloid precursor protein and presenilin 1
As	Arsenic
ASC	Alanine serine cysteine transporter
ASD	Autism Spectrum Disorder
As3MT	Arsenite (As3+) methyltransferase
Aβ	Amyloid Beta
BACE1	Beat secretase 1 (beta-site amyloid precursor protein cleaving enzyme 1)
BHMT	Betaine homocysteine methyl transferase
CBS	Cystathionine β-synthase
Cd	Cadmium
CI	Confidence interval
CNS	Central Nervous System
CTH	Cystathionine γ-lyase, also CSE (cystathionase)
Cys	Cysteine
CySSH	Thiocysteine
DATS	Diallyl trisulfide
DNA	Deoxyribonucleic acid
DMA	Dimethyl arsenic
DOPA	3,4-dihydroxyphenylalanine
GCLc	Gamma-glutamyl-cysteinyl-ligase
GPx	Glutathione peroxidase
GSH	Glutathione
GSSG	Glutathione oxidized
H_2_S	Hydrogen sulfide
Hcy	Homocysteine
HD	Huntington Disease
Hg	Mercury
IFNy	Interferon gamma
IL1β	Interleukin 1 beta
IQR	Interquartile range
KO	Knock out
MeHg	Methylmercury
Met	Methionine
MMA	Monomethyl arsenic
MAT	Methionine adenosyl transferase
MAT1	Methionine adenosyl transferase isoform type 1
MAT2	Methionine adenosyl transferase isoform type 2
MAT2A	Methionine adenosyl transferase subunit 2A (catalytic)
MAT2b	Methionine adenosyl transferase subunit 2Bb (regulatory)
MPR1	Multidrug resistance-associated protein 1
MS	Multiple Sclerosis
MTR	Methionine synthase
MTHFR	Methyl tetrahydrofolate reductase
NaHS	Sodium hydrosulfide
NMDA	N-methyl-D Aspartate
NR2A	NMDA receptor subunit 2A
NR2B	NMDA receptor subunit 2B
Pb	Lead
PD	Parkinson Disease
ROS	Reactive Oxygen Species
RNA	Ribonucleic acid
RNS	Reactive Nitrogen Species
RSS	Reactive Sulfur Species
SAH	S-adenosyl-homocysteine
SAM	S Adenosyl methionine
SD	Standard deviation
SCAA	Sulfur-containing amino acid
SE	Standard error
SEM	Standard error of the mean
-SH	Sulfhydryl group/thiol group
SLC1	Solute carrier family 1
SLC1A1	Solute carrier family 1 member 1, also EAAC1 or EAAT3 (excitatory amino acid carrier or transporter)
SLC1A4	Solute carrier family 1 member 1, also ASCT1 (alanine serine cysteine transporter 1)
SLC6	Solute carrier family 6
SLC6A6	Solute carrier family 6 member 6, also TauT (taurine transporter)
SLC7	Solute carrier family 7
SLC7A5	Solute carrier family 7 member 5, also LAT1 (large amino acid transporter 1)
SLC7A6	Solute carrier family 7 member 6
SLC7A8	Solute carrier family 7 member 6, also LAT2 (large amino acid transporter 2)
SLC7A11	Solute carrier family 7 member 6, also xCT (glutamate cystine transporter)
SNP	Single nucleotide polymorphism
THF	Tetrahydrofolate
TNFα	Tumor necrosis factor alpha
TSP	Transsulfuration pathway
XAG	Aspartic acid-glutamate transport system
Xc	Cystine system
