# Supplementary material for: Sulfur-Containing Amino Acid Homeostasis in the Central Nervous System: From Physiology Regulation to Metal-Induced Neurotoxicity
Source: Metabolites. 2026 Jul 1;16(7):461. doi: 10.3390/metabo16070461 (PMC13413942; doi:10.3390/metabo16070461)
Supplement: Supplementary file 1 [file metabolites-16-00461-s001.zip › metabolites-4371564-supplementary.pdf]

Supplementary Materials

SECTION S1.2.2.

Genes involved in SCAA homeostasis are differentially expressed across the brain.

Single-cell RNA-seq analysis

To examine the expression of genes involved in sulfur-containing amino acid (SCAA) homeostasis during brain development, we analyzed publicly available single-cell RNA sequencing data from mouse brain samples deposited under GEO accession GSE133531 [1]. We focused on embryonic day 15.5 (E15.5) and postnatal days 0 and 3 (P0 and P3), as these stages cover relevant transitions in early CNS maturation.

Raw count matrices were uploaded to the Trailmaker™ platform (Parse Biosciences; https://app.trailmaker.parsebiosciences.com/), where data processing and visualization were performed. The analysis was completed on November 27, 2025. Quality control was carried out separately for each sample. Cells with fewer than 2,000 detected transcripts were excluded to reduce background signal. Barcodes with mitochondrial transcript content above 10 % were removed, as they likely represented damaged or dying cells. Additional outliers were identified from the relationship between the number of detected genes and total transcript counts using linear regression, applying a significance threshold of $p<0.001$. Potential doubles were detected with scDblFinder and filtered using sample-adjusted probability thresholds ranging from 0.38 to 0.50.After quality control, high-quality cells were normalized using the LogNormalize approach implemented in Seurat. Principal component analysis was used for dimensionality reduction, and datasets from the different developmental stages were integrated with Harmony to reduce batch-related variation. Cell clustering was performed using the Leiden algorithm, and the resulting structure was visualized with Uniform Manifold Approximation and Projection (UMAP).

Cluster markers were identified by comparing each cluster against all other cells using the Wilcoxon rank-sum test implemented in the presto package. Cell identities were assigned with ScType mouse brain reference database [2]. These annotated clusters were then used to evaluate the distribution of SCAA-related genes across developmental stages and major brain cell populations.

Data Acquisition, selection and analysis of GTEx samples

Gene expression RNA-seq data (RSEM TPM) and clinical data from GTEx project samples [3] (version gtex_v10) were accessed through the GTEx Portal API using the R package gtexr [4]. Only samples from the Brain-cortex (n=270), Brain-hippocampus (n=255), Brain-putamen (basal ganglia) (n=254), and Brain – cerebellum (n=266) tissues were included in the analysis, together with age information categorized into six standard 10-year age brackets.

Selection of Gene Expressions: Genes corresponding to SCAA transporter (*Slc1a1, Slc1a4, Slc7a5, Slc7a6, Slc7a8, and Slc7a11*) and SCAA metabolism (*Mat1a, Mat2a, Mat2b, Ahcy, Mtr, Bhmt, Mthfr, Cbs and Cth*) were selected for the analysis considering their relevance in SCAA homeostasis as described in Section 1.2

Statistical Analysis: All TPM values were transformed using the $\log_2(\text{tpm}+1)$ transformation. The Kruskal–Wallis test and Dunn's post hoc test for multiple pairwise comparisons were used to evaluate differences in gene expression among brain tissues of interest (cortex, hippocampus, putamen, and cerebellum). Additionally, exploratory analyses of pairwise associations between the expression of each gene and age were conducted using Spearman's rank correlation coefficient. A *p*-value less than 0.05 was considered statistically significant, and all analyses were performed using R version 4.3.1 (R Foundation for Statistical Computing, Vienna, Austria) [5].

1. Jessa, S.; Blanchet-Cohen, A.; Krug, B.; Vladoiu, M.; Coutelier, M.; Faury, D.; Poreau, B.; De Jay, N.; Hebert, S.; Monlong, J.; et al. Stalled developmental programs at the root of pediatric brain tumors. *Nat Genet* **2019**, *51*, 1702-1713, doi:10.1038/s41588-019-0531-7.
2. Ianevski, A.; Giri, A.K.; Aittokallio, T. Fully-automated and ultra-fast cell-type identification using specific marker combinations from single-cell transcriptomic data. *Nat Commun* **2022**, *13*, 1246, doi:10.1038/s41467-022-28803-w.
3. Consortium, G.T. The Genotype-Tissue Expression (GTEx) project. *Nat Genet* **2013**, *45*, 580-585, doi:10.1038/ng.2653.
4. Warwick, A.N.; Zuckerman, B.; Ung, C.Y.; Luben, R.; Olvera-Barrios, A. gtexr: A convenient R interface to the Genotype-Tissue Expression (GTEx) Portal API. *The Journal of Open Source Software* **2025**, *10*, 8249, doi:10.21105/joss.08249.
5. R Core Team (2023). _R: A Language and Environment for Statistical Computing_. R Foundation for Statistical Computing, Vienna, Austria. <https://www.R-project.org/>.

SECTION S3.2

Toxic metals disrupt sulfur amino acid homeostasis

For Section 3.2 *Toxic metals disrupt sulfur amino acid homeostasis*, two authors (WLGA and GIVC) independently searched the Web of Science (WoS) Core Collection and Pubmed databases to identify relevant studies published up to December 2025, with no restriction on the initial publication date. The research strategy combined one sulfur-containing amino acid SCAA-related term with one metal exposure-related term. The SCAA-related terms were: hydrogen sulfide, cysteine, homocysteine, cystathionine, methionine, cystine, H_2S, and transsulfuration AND metal exposure-related terms were: arsenic exposure, Pb exposure, cadmium exposure, mercury exposure, and aluminum exposure. Metal exposure terms were restricted to the title and abstract fields. This search retrieved 1159 records from WoS and 250 records from PubMed.

Selection of studies: Studies were screened according to predefined eligibility criteria. We included peer-reviewed original articles written in English that reported quantitative data from human studies or rodent/non-human primate experimental models. Eligible studies were required to evaluate the effect of metal exposure on SCAA homeostasis. Review articles, studies without explicit quantitative data, and articles not directly addressing SCAA-related outcomes were excluded. We also excluded studies focused primarily on supplementation with SCAAs or related compounds as protective interventions against metals toxicity, as well as studies using acute or subchronic exposure models.

Title and abstract screening was performed independently by two authors (WLGA and GIVC). Full-text evaluation was then conducted for potentially eligible articles to confirm inclusion. Disagreements were resolved by re-reading the article and jointly reassessing the data; when needed, a senior author arbitrated the final decision. The eligible studies were ultimately 66.

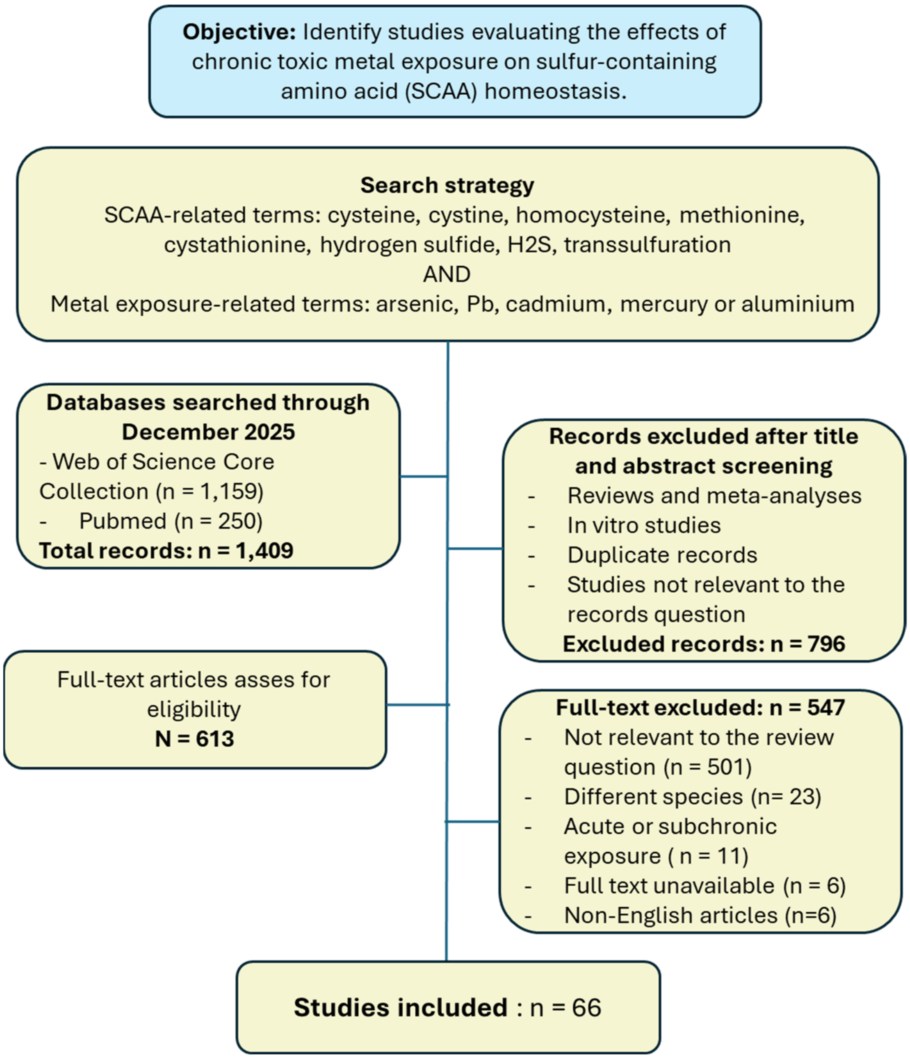

Supplementary Figure S1. Workflow describing the literature search and study selection process for section 3.2: Toxic metals disrupt sulfur amino acid homeostasis.
